# The Washington Meeting

**Published:** 1887-07

**Authors:** 


THE WASHINGTON MEETING.

The members of the local committee having in charge the arrangements for the sessions of the Section of Dental and Oral Surgery in the coming Congress are making excellent progress. They have secured admirable accommodations for both the section meetings and the working and practical departments. The former will be held in a church that is capable of comfortably seating a thousand persons, while the latter will be in the Franklin School Building, and both these are within two blocks of the Arlington Hotel, which will be the headquarters of the Section.

All committees of the Section are making satisfactory advancement, and a superabundance of papers is already secured. In the general Executive Committee of the Congress it is freely admitted that the present probabilities are that the Section of Dental and Oral Surgery will take a prominent position, if it be not the most successful of the Sections of the Congress. It should be recollected, however, that a success is not to be secured by mere numbers. A mob is not an army, and a mere aggregation of dentists will not make a good section. We are to be estimated by the scientific work we accomplish, and not by the count of noses.

There must be no crude work done, or unscientific theories or methods presented. More men will be needed to apply the gag than to give the spur. We have the reputation of dealing largely in rhetoric, and for making speeches that serve only to amuse and entertain. Scientific men do not mistake sound for sense, and we hope for the sake of our character abroad that if the fool-killer must make his rounds it will be just before the Congress meets, and that he will snatch away our professional speech-makers, leaving only the practical, sound, level-headed and modest workers, who do not get their theories at second-hand, and who are ready with facts instead of fancies.

